# Risk factors and outcomes of incidental parathyroidectomy in thyroidectomy: A systematic review and meta-analysis

**DOI:** 10.1371/journal.pone.0207088

**Published:** 2018-11-09

**Authors:** Binglong Bai, Zhiye Chen, Wuzhen Chen

**Affiliations:** 1 Department of General Surgery (Thyroid Center), Second Affiliated Hospital, School of Medicine, Zhejiang University, Hangzhou, Zhejiang, P. R. China; 2 Cancer Institute, Key Laboratory of Cancer Prevention and Intervention, China National Ministry of Education, Key Laboratory of Molecular Biology in Medical Sciences, Hangzhou, Zhejiang Province, China; 3 Department of Surgical Oncology, Second Affiliated Hospital, School of Medicine, Zhejiang University, Hangzhou, Zhejiang, P. R. China; University of Manitoba, CANADA

## Abstract

**Introduction:**

Postoperative hypocalcemia is the most common complication of thyroidectomy. Incidental parathyroidectomy (IP) was thought to be associated with postoperative hypocalcemia. However, according to previous studies, the risk factors and clinical outcomes of IP remain controversial.

**Methods:**

Eligible studies were searched in databases including PubMed, Web of Science, and EMBASE from January 1990 to September 2017. Articles focusing on the relationship between IP and postoperative hypocalcemia were included. The risk of publication bias was assessed using Begg’s test and Egger’s regression asymmetry test. Pooled analysis was performed to evaluate the effect of IP on postoperative hypocalcemia and related risk factors. Sensitivity analysis was also conducted to test the stability of our results. The effects of hypocalcemia type, permanent definition, IP incidence, total thyroidectomy, and malignancy operation were also examined using a further subgroup analysis.

**Results:**

Thirty-five studies were finally included in the analysis after an exhaustive literature review. Pathology data demonstrate that incidental parathyroidectomy occurred in various locations: intrathyroidal (2.2–50.0%), intracapsular (16.7–40.0%) and extracapsular (15.7–81.1%) regions. Overall, the analysis found that malignancy (RR = 1.60, 95% CI: 1.27 to 2.02; p< 0.0001), central neck dissection (RR = 2.35, 95% CI: 1.47 to 3.75; p = 0.0004), total thyroidectomy (RR = 1.42, 95% CI: 1.20 to 1.67; p< 0.0001) and reoperation (RR = 1.81, 95% CI: 1.20 to 2.75; p = 0.005) were significant risk factors of IP in thyroid surgery. There was an obvious effect of IP on temporary/permanent (RR = 1.59, 95% CI: 1.37 to 1.84; p< 0.0001) and permanent (RD = 0.0220, 95% CI: 0.0069 to 0.0370; p = 0.0042) postoperative hypocalcemia. Sensitivity analysis showed that these results were robust. The subgroup analysis found that IP played a significant role in both biochemical and clinical hypocalcemia in thyroidectomy (p < 0.0001 and p = 0.0003, separately). The association of IP and permanent hypocalcemia using different definitions (6 months or more than 12 months) was also confirmed by the analysis. IP increased the incidence of temporary/permanent and permanent hypocalcemia for cases undergoing total thyroidectomy (40.4% vs 24.8% and 5.8% vs 1.4%, respectively). Thyroidectomy with IP was associated with more permanent hypocalcemia (RR = 3.10, 95% CI: 2.01 to 4.78; p< 0.0001) in malignant cases but was not associated with temporary/permanent hypocalcemia.

**Conclusions:**

Malignancy, central neck dissection, total thyroidectomy and reoperation were found to be significant risk factors of IP. IP increases the risk of postoperative hypocalcemia after thyroidectomy. We recommend a more meticulous intraoperative identification of parathyroid gland in thyroidectomy to reduce IP, particularly for total thyroidectomy and malignancy cases.

## Introduction

Thyroidectomy is a widely used and relatively safe procedure having a major postoperative morbidity of less than 5% for experienced surgeons.[1] Major complications include recurrent laryngeal nerve injury, postoperative hemorrhage and hypocalcemia.[2] Among these, hypocalcemia is the most common complication, with an incidence of 7–51%.[3–5] The quality of life is decreased in patients suffering from postoperative hypocalcemia, particularly in those suffering from clinical hypocalcemia. Moreover, prolonged or long-term calcium supplementation might be necessary for transient or permanent hypocalcemia. Surgical trauma, devascularization, extent of surgery, and incidental parathyroidectomy (IP) have been found to affect postoperative hypocalcemia.[2, 4, 6]

IP has been defined as parathyroid tissue found in a postoperative specimen and occurs in 6–28% of thyroidectomy.[3–5] Even with careful dissection and sufficient anatomical knowledge, the identification and in situ preservation of parathyroid tissue remains challenging. There are several anatomic variants of parathyroid glands, particularly of the inferior parathyroid.[7] Parathyroid glands are usually surrounded by fat and connective tissue, and this can be excised as thyroid, lymph or fat tissue. Some studies have shown that pathology type, procedure extent, reoperation and central neck dissection are risk factors of IP in thyroidectomy.[4, 8] However, these relationships have not been found in all studies.[9–11]

The influence of IP on postoperative hypocalcemia remains controversial. Some studies have found an increased risk of postoperative hypocalcemia in IP patients, particularly of biochemical hypocalcemia.[4, 11, 12] However, other studies have found no significant relationship between IP and postoperative hypocalcemia.[13–15] This difference might be caused by the use of different definitions for postoperative hypocalcemia and for biochemical or clinical hypocalcemia. Further attention has been paid to permanent hypocalcemia. Most researchers have found only negative results, and this might be caused by the relatively low incidence of permanent hypocalcemia and insufficient sample sizes.[16–18] Nevertheless, some researchers have found a significant effect of IP on permanent hypocalcemia.[4, 5] Several confounding factors were involved in these results, such as the definition of permanent hypocalcemia, procedure extent, and pathology type. Based on these considerations, we conducted a systematic review and integrated related data to identify the risk factors of IP and the effect of IP on postoperative hypocalcemia in thyroidectomy. Further subgroup analyses were also performed to assess related factors that were mentioned in previous studies.

## Materials and methods

### Search strategy and selection criteria

We performed a comprehensive literature search of MEDLINE (PubMed), BIOSIS Previews (ISI Web of Knowledge) and the Cochrane library from January 1990 to September 2017. We also manually added relevant articles by reviewing the references. Search terms included: "incidental parathyroidectomy" or "unintentional parathyroidectomy" or "inadvertent parathyroidectomy", "thyroidectomy" or "thyroid surgery" or "thyroid resection", and "hypocalcemia" or "hypocalcemia" or "hypoparathyroidism". The studies included were confined to randomized controlled trials and case control studies and cohort studies with convertible effects data of IP in thyroidectomy. Studies that did not define postoperative hypocalcemia or permanent hypocalcemia were excluded. For postoperative hypocalcemia, data that were collected with a clear distinction between biochemical and clinical hypocalcemia were included. Most of the included papers only defined hypocalcemia and permanent hypocalcemia and did not define transient hypocalcemia. For this reason, transient hypocalcemia is not discussed in this study. We defined temporary/permanent hypocalcemia as any hypocalcemia occurring after thyroidectomy, regardless of hypocalcemia duration, to ensure that we could present and discuss the data appropriately. For permanent hypocalcemia, we only included data for which permanent hypocalcemia was defined as lasting more than 6 months, consistent with relevant studies.[4, 18] If several papers extracted data from the same population, we only included the most recent. Paper selection involved four steps, including identification, screening, the determination of eligibility and inclusion. For identification, we found papers through searching the databases, and duplicate papers were determined based on title, author and abstract. There was no disagreement in this step. At the screening and eligibility steps, some disagreement existed among us, with Kappa values of 0.8922 and 0.8256.

### Data extraction and quality assessment

Data were independently extracted by two authors (BL Bai and WZ Chen). Detailed information was recorded on first author, year of publication, location, study type, pathology type, procedure extent, biochemical or clinical hypocalcemia, definition of permanent hypocalcemia, the number and location of parathyroid glands found in the specimen, the number of case and control groups, the event of malignancy, central neck dissection, reoperation, total thyroidectomy, temporary/permanent and permanent hypocalcemia, and Newcastle-Ottawa Scale score (NOS). The Newcastle-Ottawa Scale for Quality was used to assess the included cohort studies, yielding a minimum score of 0 and a maximum score of 9.[19] There were three sections in the NOS; selection, comparability and outcome. We modified some features in the sections relating to comparability and outcome. For the section on comparability, one score was used for study controls for basic characters, such as age, gender and histopathology. Another score was used for study controls for the additional factor of surgery extent. For the outcome section, one score was used for a study follow-up of more than 6 months. We considered a follow-up in which fewer than 10% of the cohort was lost as small and unlikely to introduce bias.

### Statistical methods

Meta-analysis was performed under the R environment 3.4.1 in the Meta package 4.8–3.[20] In accordance with recommendations of the Cochrane Collaboration, risk ratio (RR), risk difference (RD) and confidence interval (CI) were calculated using the random-effects model using an inverse variance weight to compare the difference.[21–23] If zero events happened in both the experimental and control groups, the pooled RD estimates and 95% CIs were applied. Otherwise, RR was applied. RR below one or RD below zero indicates a benefit of the experimental intervention. We estimated 95% CI from the 2.5th and 97.5th percentiles of the posterior distribution and calculated two-sided p values from the posterior distribution. If the 95% CI does not contain the valid value 0 for RD or 1 for RR, the result is considered to represent a significant difference. Publication bias was assessed using funnel plots, Begg’s test and Egger’s regression asymmetry test.[24] The heterogeneity between trials was estimated from the median between-trial variance (τ^2^) observed in the posterior distribution. Meta-regression analysis was conducted to assess the causes of heterogeneity.[25]

## Results

### Description of studies

**Fig 1:**
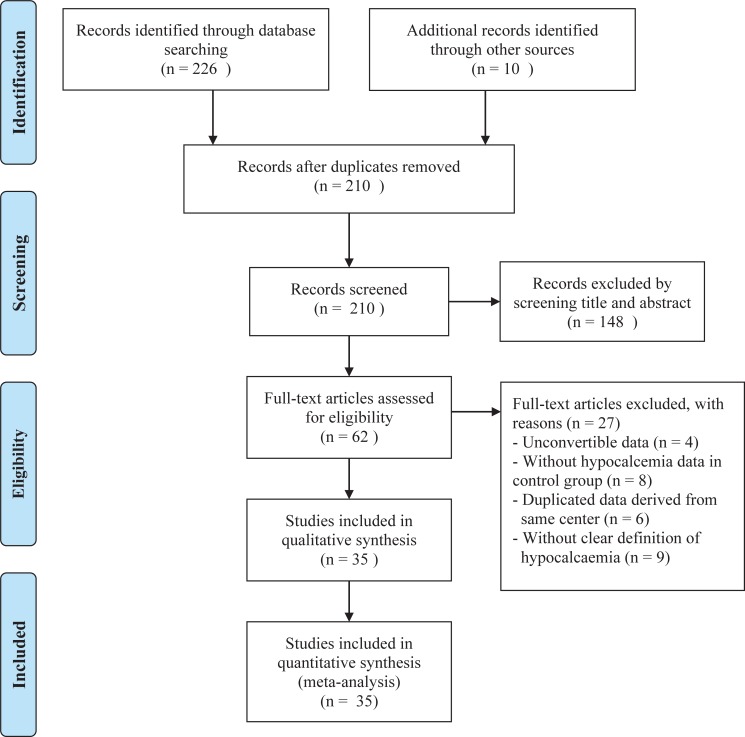
Flow diagram of the study selection. Two authors (BL Bai and WZ Chen) participated separately in the entire study selection process. We identified papers through a database search, and duplicate papers were identified based on title, author and abstract. There was no disagreement at this step. For screening and eligibility, some disagreement occurred, with Kappa values of 0.8922 and 0.8256.

A total of 236 articles were identified through the literature search. After duplicates were removed, 210 articles were included for screening. Among these, 62 eligible full-text articles were selected after carefully checking the titles and abstracts.(Fig 1) However, we excluded 27 of the 62 articles because they contained unconvertible data (4 articles)[26–29], lacked hypocalcemia data for the control group (8 articles)[30–37], duplicated data derived from same center (6 articles), or did not give a clear definition of hypocalcemia (9 articles)[38–46]. Finally, 35 articles were included in the systematic review and meta-analysis.[3–5, 8–18, 47–67] Details on first author, year of publication, location, study type, pathology, procedure extent, the definition of permanent hypocalcemia and NOS score were recorded. (Table 1) Two prospective cohorts and 33 retrospective cohorts were included in our study. The pathology was divided into benign and malignant cases. Two studies included only benign cases, 7 studies included only malignant cases, and the remaining 26 studies included both benign and malignant cases. The procedures used in the included studies were diverse. Twelve studies focused their discussion on total thyroidectomy. Twenty-three studies defined permanent hypocalcemia as having a duration of more than 6 months. The NOS of the included studies ranged from 6 to 9, suggesting that the studies were of acceptable quality. The main characteristics of the studies that were excluded based on the full-text article assessment are shown in S1 Table.

**Table 1:** Main characteristic of included studies.

Author	Year	Location	Study type	Pathology	Procedure extent	Biochemical hypocalcemia definition	Clinical hypocalcemia definition	Permanent measurement	Permanent duration	NOS score
Sasson[62]	2001	USA	RC	Benign & Malignant	TT, ST, LB	NS	Symptom*	Symptom*	12 months	8
Sakorafas[61]	2005	Greece	RC	Benign & Malignant	TT, NT, LB, ST	sCa<8.0mg/dL	Symptom*	Symptom*	12 months	7
Gourgiotis[13]	2006	Greece	RC	Benign & Malignant	TT, NT, LB	sCa<8.0mg/dL	NS	NS	-	7
Abboud[47]	2007	Lebanon	RC	Benign & Malignant	TT, NT, LB	sCa<8.0mg/dL	Symptom*	NS	-	8
Cavicchi[49]	2007	Italy	PC	Benign & Malignant	UT, BT	NS	Symptom*	NS	-	6
Irkorucu[51]	2007	Turkey	RC	Benign & Malignant	TT, NT, LB, ST	NS	Symptom*	NS	-	7
Page[14]	2007	France	RC	Benign only	TT	sCa<8.0mg/dL	SNS	sCa<8.0mg/dL	24 months	7
Sippel[64]	2007	USA	RC	Benign & Malignant	TT, CT, ST, LB	sCa<8.0mg/dL	Symptom*	NS	-	7
Manouras[54]	2008	Greece	RC	Benign & Malignant	TT	sCa<8.5mg/dL	NS	NS	-	9
Erbil[50]	2009	Turkey	RC	Benign only	TT, NT	sCa<8.0mg/dL	NS	NS	-	8
Rajinikanth[60]	2009	India	RC	Benign & Malignant	TT, CT	sCa<8.0mg/dL	NS	Medication^#^	6 months	7
Sorgato[8]	2009	Italy	RC	Benign & Malignant	TT, CT, ST, LB	sCa<8.4mg/dL	NS	Medication^#^	12 months	8
Turanli[66]	2009	Turkey	RC	Malignant only	TT, ST, CT	NS	NS	Medication^#^	6 months	6
Ondik[56]	2010	USA	RC	Malignant only	CT	NS	Symptom*	Medication^#^	6 months	8
Spiliotis[3]	2010	Greece	RC	Benign & Malignant	TT, ST	sCa<8.0mg/dL	NS	NS	-	7
Youssef[9]	2010	Egypt	RC	Benign & Malignant	UT, BT	sCa<8.0mg/dL	Symptom*	NS	-	8
Khairy[10]	2011	Saudi Arabia	RC	Benign & Malignant	TT, CT, ST, LB	sCa<8.0mg/dL	NS	sCa<8.0mg/dL	6 months	8
Qasaimeh[59]	2011	Jordan	RC	Benign & Malignant	TT, ST, LB	sCa<8.0mg/dL	NS	Medication^#^	6 months	8
Kalyoncu[52]	2013	Turkey	RC	Benign & Malignant	TT, NT, ST	sCa<8.0mg/dL	NS	Medication^#^	6 months	6
Nair[55]	2013	India	RC	Benign & Malignant	TT	sCa<8.0mg/dL	NS	Medication^#^	6 months	7
Paek[12]	2013	Korea	RC	Malignant only	TT	NS	Symptom*	Medication^#^	12 months	7
Sheahan[63]	2013	Ireland	PC	Benign & Malignant	TT	sCa<8.0mg/dL	Symptom*	Medication^#^	6 months	7
Del Rio[16]	2014	Italy	RC	Benign & Malignant	TT	NS	NS	Medication^#^	12 months	6
Pergel[58]	2014	Turkey	RC	Benign & Malignant	TT	NS	NS	sCa<8.0mg/dL	6 months	7
Prazenica[17]	2014	Czech Republic	RC	Benign & Malignant	TT, CT, LB	sCa<8.0mg/dL	NS	Medication^#^	12 months	8
Song[65]	2014	Korea	RC	Malignant only	TT	sCa<8.0mg/dL	NS	Medication^#^	6 months	9
Lee[53]	2015	Korea	RC	Malignant only	TT	sCa<8.0mg/dL	Symptom*	NS	-	6
Wang[67]	2015	China	RC	Benign & Malignant	TT	sCa<8.8mg/dL	NS	Medication^#^	12 months	6
Applewhite[48]	2016	USA	CC	Benign & Malignant	TT	sCa<8.2mg/dL	Symptom*	NS	-	9
Manatakis[11]	2016	Greece	RC	Benign & Malignant	TT	sCa<8.0mg/dL	Symptom*	Medication^#^	6 months	9
Ozemir[57]	2016	Turkey	RC	Benign & Malignant	TT, CT, LB	sCa<8.5mg/dL	NS	Medication^#^	12 months	6
Zhou[18]	2016	USA	RC	Benign & Malignant	TT, CT, LB	NS	NS	Medication^#^	6 months	8
Du[15]	2017	China	RC	Malignant only	TT+CND	NS	Symptom*	NS	-	8
Lin[4]	2017	Taiwan	RC	Benign & Malignant	TT, ST, LB	sCa<8.0mg/dL	NS	Medication^#^	6 months	8
Sitges-Serra[5]	2017	Spain	PC	Malignant only	TT+CND	sCa<8.0mg/dL	NS	Medication^#^	12 months	9

Abbreviation: RC, retrospective cohort; PC prospective cohort; TT total thyroidectomy; ST subtotal thyroidectomy; CT completion thyroidectomy; LB lobectomy; NT near-total thyroidectomy; CND central neck dissection; UT unilateral thyroidectomy; BT bilateral thyroidectomy; NOS, Newcastle-Ottawa Scale; sCa serum calcium level; PTH

* Symptom: hypocalcemic Symptom* including paraesthesia, muscle cramping, perioral numbness or tingling.

^#^ Medication: medication requirement of calcium and/or vitamin D.

The distribution of IP among the different high-risk groups is shown in Table 2. Data regarding malignancy were available in 18 studies, data on central neck dissection were available in 12 studies, data on reoperation were available in 4 studies, and data on total thyroidectomy were available in 12 studies. In total, 2033 IP relating to 16423 cases were included in our analysis, with an average incidence of 12.4%. (S2 Table) Regarding temporary/permanent postoperative hypocalcemia, data on biochemical hypocalcemia were available in 22 studies, and data on clinical hypocalcemia were available in 14 studies. Among these studies, 8 exhibited both biochemical and clinical hypocalcemia.

**Table 2:** The distribution of IP risk factors.

Author	Year	Case, n		Malignancy, n		Central neck dissection, n		Reoperation, n		Total thyroidectomy, n	
		IP	Non-IP	IP	Non-IP	IP	Non-IP	IP	Non-IP	IP	Non-IP
Sasson[62]	2001	21	120	11	62	5	10	-	-	12	57
Sakorafas[61]	2005	28	130	4	40	-	-	-	-	-	-
Gourgiotis[13]	2006	68	247	10	65	-	-	-	-	-	-
Abboud[47]	2007	38	269	9	54	3	13	-	-	13	90
Irkorucu[51]	2007	10	263	2	13	-	-	-	-	-	-
Sippel[64]	2007	33	480	17	120	3	14	3	14	14	110
Manouras[32]	2008	100	408	16	66	-	-	-	—	-	-
Erbil[50]	2009	48	392	-	-	-	-	-	-	14	97
Rajinikanth[60]	2009	47	317	30	120	13	41	-	-	-	-
Sorgato[8]	2009	70	812	37	203	28	141	-	-	64	610
Turanli[66]	2009	25	392	-	-	-	-	14	92	-	-
Ondik[56]	2010	13	29	-	-	5	21	-	-	-	-
Spiliotis[3]	2010	32	283	14	21	-	-	-	-	18	83
Youssef[9]	2010	26	181	9	26	-	-	-	-	16	108
Khairy[10]	2011	47	240	19	104	2	15	-	-	30	61
Qasaimeh[59]	2011	20	213	6	16	-	-	-	-	8	97
Prazenica[17]	2014	58	1010	7	96	7	64	-	-	52	704
Song[65]	2014	90	364	-	-	86	246	-	-	-	-
Applewhite[48]	2016	283	271	197	158	80	20	-	-	-	-
Manatakis[11]	2016	70	211	23	63	-	-	-	-	-	-
Zhou[18]	2016	78	308	37	43	25	15	4	21	56	185
Lin[4]	2017	204	2982	80	540	40	73	28	255	89	567

Abbreviation: IP incidental thyroidectomy.

### Publication bias, sensitivity analysis and heterogeneity assessment

Begg’s and Egger’s test were conducted to assess publication bias in this meta-analysis. In the risk factor analysis, all tests found no significance for malignancy, central neck dissection, or total thyroidectomy. For the analysis of reoperation, the number of studies was too small to conduct Begg’s or Egger’s test. None of the tests showed significance for temporary/permanent postoperative hypocalcemia (Begg’s p = 0.4769 and Egger’s p = 0.253) or permanent hypocalcemia (Begg’s p = 0.6534 and Egger’s p = 0.1139). The funnel plot of temporary/permanent postoperative hypocalcemia and permanent hypocalcemia was somewhat asymmetric, indicating some publication bias in the analysis. (S1 and S2 Figs) The meta-analysis was recalculated after omitting every record included in the analysis to check the sensitivity of our overall results. As shown in S3 and S4 Figs, the results for temporary/permanent and permanent hypocalcemia were robust after omitting each of the records in turn. Three variables were included in the meta-regression analysis; continent, year of study and NOS. We found that all three factors did not have any impact on the findings and did not explain the observed heterogeneity in the analysis. Only the continent of Europe might partly explain the heterogeneity found for permanent hypocalcemia, having a p value of 0.0898. (S3 Table) Further analysis of European studies found no significant effect for permanent hypocalcemia (RD = 0.0058; 95% CI: -0.0121 to 0.0236; p = 0.5261). Another analysis of non-European articles showed that IP had a significant effect on permanent hypocalcemia (RD = 0.0307; 95%CI: 0.0116 to 0.0497; p = 0.0016).

### Pathology results

Detailed pathology data are listed in S4 Table. Only one parathyroid gland was incidentally excised in the majority of IP cases, while two or more parathyroid glands were found postoperatively in the remaining 6.4–20.0% of cases. The location of parathyroid gland in the specimens was diverse. In total, 2.2–50.0% of parathyroid glands were identified in an intrathyroidal location, 16.7–40.0% were identified in an intracapsular location, and 15.7–81.1% were identified in an extracapsular location. (S4 Table)

### Risk factors of IP

To identify the risk factors of IP, we collected IP incidence data by examining factors such as malignancy, central neck dissection, total thyroidectomy and reoperation. (Table 3) The risk of IP in thyroidectomy was significantly higher for malignancy cases (RR = 1.60; 95% CI: 1.27 to 2.02; p< 0.0001). Central neck dissection was found to be associated with higher risk of IP in thyroidectomy (RR = 2.35; 95% CI: 1.47 to 3.75; p = 0.0004). The rate of total thyroidectomy in IP (57.2%) was obviously higher than that in non-IP (38.0%) cases. Four studies reported data on reoperation rate. Meta-analysis of these data showed that reoperation influenced the incidence of IP in thyroidectomy (RR = 1.81; 95% CI: 1.20 to 2.75; p<0.005).

**Table 3:** Risk factors of IP.

Risk factors	IP, n		Non-IP, n		RR [95% CI]	p value	Number of studies
	Events	Cases	Events	Cases			
Malignancy	528	1233	1810	8745	1.60 [1.27; 2.02]	< 0.0001	18
Central neck dissection	297	982	673	7202	2.35 [1.47; 3.75]	0.0004	12
Total thyroidectomy	386	675	2769	7290	1.42 [1.20; 1.67]	< 0.0001	12
Reoperation	49	340	382	4162	1.81 [1.20; 2.75]	0.005	4

Abbreviation: IP, incidental thyroidectomy; RR, risk ratio; CI, confidence interval.

### Temporary/permanent postoperative hypocalcemia

**Fig 2:**
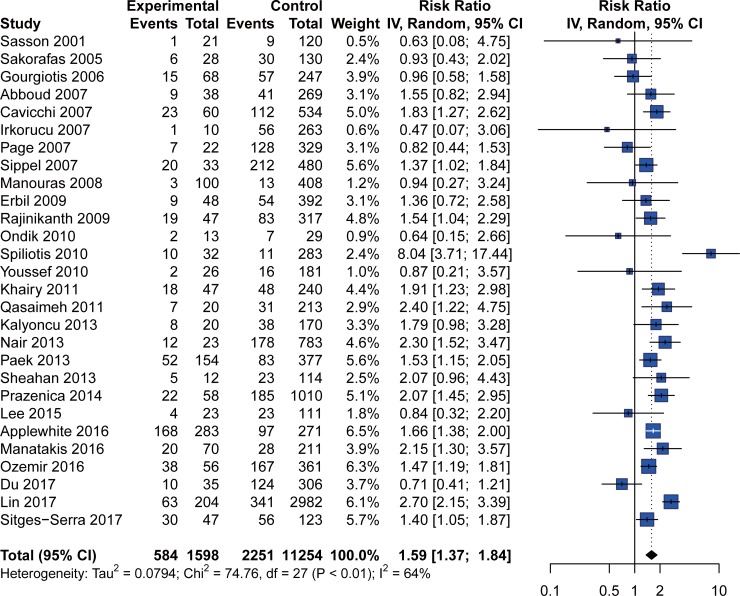
Forest plot of temporary/permanent postoperative hypocalcaemia.

Temporary/permanent postoperative hypocalcemia data were available in 28 studies after excluding studies without a clear hypocalcemia definition. When calculating the overall effect, we pooled all data regardless of whether the hypocalcemia was biochemical or clinical. For 8 studies containing both biochemical and clinical hypocalcemia, we preferentially selected biochemical hypocalcemia for the overall effect analysis. The overall analysis found a significantly higher risk of temporary/permanent postoperative hypocalcemia in IP cases (Fig 2), with an RR of 1.59 (95% CI: 1.37 to 1.84; p< 0.0001). Subgroup analysis was performed according to factors of concern (Table 4). An analysis only including biochemical hypocalcemia showed that IP was associated with a higher risk of postoperative hypocalcemia (RR = 1.70; 95% CI: 1.45 to 1.99; p< 0.0001). Fourteen studies reported data on clinical hypocalcemia. Further subgroup analysis found that IP increased the risk of clinical hypocalcemia in thyroidectomy with an RR of 1.54 (95% CI: 1.22 to 1.94; p = 0.0003). To study the influence of IP incidence on hypocalcemia, we divided all data into two groups according to the average IP incidence of 12.4% (>12.4% and < = 12.4%). The analysis showed a significant result in both groups. Total thyroidectomy and malignancy were found to be high risk factors of IP. A specific analysis of cases undergoing total thyroidectomy confirmed the influence of IP on temporary/permanent postoperative hypocalcemia. Data on malignancy were reported in only 5 studies. A comparable incidence of temporary/permanent hypocalcemia was identified between IP and non-IP cases.

**Table 4:** Subgroup analysis of hypocalcaemia type, permanent definition, IP incidence, total thyroidectomy and malignancy.

Subgroups		IP, n		Non-IP, n		RR [95% CI] or RD [95% CI]	p value	Number of studies
		Events	Cases	Events	Cases			
Temporary/permanent hypocalcemia, hypocalcaemia type								
	Biochemical	495	1305	1860	9625	1.67 [1.43; 1.96]	< 0.0001	22
	Clinical	154	806	494	3396	1.54 [1.22; 1.94]	0.0003	14
Permanent hypocalcaemia, duration of permanent definition								
	6 months	45	672	142	6556	0.0243 [0.0001; 0.0486]	0.0495	13
	≥12 months	35	605	76	4089	0.0200 [0.0007; 0.0393]	0.0422	10
Incidence of IP < 12%								
	Total hypocalcaemia	197	577	1493	7859	1.79 [1.36; 2.36]	< 0.0001	13
	Permanent hypocalcaemia	35	503	163	7750	0.0163 [-0.0122; 0.0449]	0.2626	11
Incidence of IP ≥ 12%								
	Total hypocalcaemia	387	1021	758	3395	1.51 [1.36; 1.66]	< 0.0001	15
	Permanent hypocalcaemia	45	774	55	2895	0.024 [0.0075; 0.0405]	0.0044	12
Total thyroidectomy								
	Total hypocalcaemia	311	769	753	3033	1.45 [1.17; 1.80]	0.0007	10
	Permanent hypocalcaemia	34	590	50	3561	0.0201 [0.0006; 0.0397]	0.0429	10
Malignancy								
	Total hypocalcaemia	98	272	293	946	1.16 [0.84; 1.60]	0.3628	5
	Permanent hypocalcaemia	32	329	54	1285	3.10 [2.01; 4.78]	< 0.0001	5

Abbreviation: IP incidental thyroidectomy; RR, risk ratio; RD, risk difference; CI, confidence interval.

### Permanent hypocalcemia

**Fig 3:**
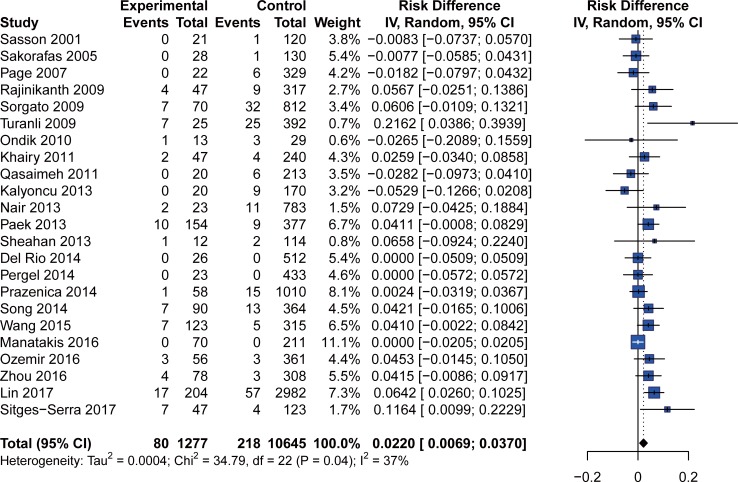
Forest plot of permanent postoperative hypocalcaemia.

Due to the low incidence of permanent hypocalcemia, the number of events in several studies was zero for both IP and non-IP. We introduced RD to identify the difference between two groups in accordance with the recommendations of the Cochrane Collaboration. As shown in Fig 3, the overall analysis of 23 studies found that IP was significantly associated with permanent hypocalcemia (RD = 0.0220; 95% CI: 0.0069 to 0.0370; p = 0.0042). Considering the different definitions of permanent hypocalcemia, we performed separate analyses of cases with definitions of permanent hypocalcemia as being 6 months or longer than 12 months (Table 3). For studies in which the definition was 6 months, the incidence of permanent hypocalcemia in IP (6.7%) was much higher than that in non-IP (2.2%) cases, with an RD of 0.0243 (95% CI: 0.0001 to 0.0486; p = 0.0495). For studies in which the definition of permanent hypocalcemia was hypocalcemia lasting longer than 12 months, the influence of IP on permanent hypocalcemia was confirmed (p = 0.0422). A significant association was found between IP and permanent hypocalcemia in studies with an IP incidence >12.4% but not in studies with an IP incidence of 12.4% or lower. Subgroup analysis of high risk factors found a significant effect of IP on permanent hypocalcemia in total thyroidectomy (RD = 0.0201; 95% CI: 0.0006 to 0.0397; p = 0.0429) and malignancy (RR = 3.10; 95% CI: 2.01 to 4.78; p<0.0001) cases.

## Discussion

Incidental parathyroidectomy can occur in any operation on the thyroid, even in experienced surgeons’ hands. According to the articles included in our review, the incidence of IP in thyroidectomy ranged from 2.9% to 31.0%. Among the incidentally excised parathyroid glands, 2.2–50.0% were identified as intrathyroidal postoperatively, indicating that the complete elimination of IP is almost impossible. However, more meticulous intraoperative identification of the parathyroid gland might reduce the incidence of IP because 16.7–40.0% of the incidentally excised parathyroid glands were found in an intracapsular location and 15.7–81.1% were found in an extracapsular location.

Much effort has been expended to identify the risk factors of IP in thyroidectomy. Lin et al. conducted a retrospective cohort study of 3,186 thyroidectomy cases and reported an IP incidence of 6.4%.[4] An analysis of this large set of sample data showed that malignancy, total thyroidectomy, central neck dissection and reoperation influence the incidence of IP. However, a comparable rate of IP was observed in malignant and benign cases by several researchers.[10, 11, 54, 62] We pooled these data and confirmed that malignancy is a risk factor for IP. Malignant lesions might invade the parathyroid gland along with adjacent tissue adhesions. There is a wider extent of operation in total thyroidectomy, and this increases the risk of incidental IP. The results of our meta-analysis support this hypothesis. Controversial results have been obtained concerning the effect of central neck dissection and reoperation on IP incidence.[4, 10, 54] Our meta-analysis showed that central neck dissection and reoperation significantly influence the incidence of IP.

Naturally, parathyroidectomy was considered to influence the function of parathyroid gland, regardless of whether the parathyroidectomy was intentional or incidental. Hence, the effect of IP on postoperative hypocalcemia in thyroidectomy has attracted much attention. An increased incidence of temporary/permanent postoperative hypocalcemia and permanent hypocalcemia in IP cases was confirmed by a high volume center experience.[4] However, Prazenica et al. reviewed 1,068 thyroidectomy cases and found that IP in thyroidectomy was associated only with temporary/permanent postoperative hypocalcemia and not with permanent hypocalcemia.[17] Debate continued on the association between IP and postoperative hypocalcemia.[11, 12, 14, 65] We performed a comprehensive review of the literature and meta-analysis of the results. A significant relationship was observed between IP and postoperative hypocalcemia regardless of whether the hypocalcemia was temporary/permanent or permanent.

The definitions of temporary/permanent and permanent postoperative hypocalcemia, the incidence of IP in thyroidectomy, the extent of the procedure and the final pathologic diagnoses varied, possibly accounting for the difference in the results obtained when studying this topic; thus, a further subgroup analysis was conducted. There were two different definitions of temporary/permanent postoperative hypocalcemia: a biochemical definition and a clinical definition. Although these two definitions partially overlap, the incidence of clinical hypocalcemia (4–39%) was lower than that of biochemical hypocalcemia (3–56%). [9, 15, 48, 53, 54, 65] In our study, IP was found to be associated with an increased incidence of temporary/permanent hypocalcemia, regardless of whether the biochemical or clinical definition was used. Kihara et al. followed 99 thyroidectomy cases with a detailed measure of hypocalcemia and found that serum calcium levels returned to stability after 6 months postoperatively, indicating that a definition of permanent hypocalcemia based on 6 months was suitable.[68] In accordance with this, our results found that IP cases increased the risk of permanent hypocalcemia as defined based on 6 months and more than 12 months. The overall incidence of IP in all the included studies was 12%. According to the overall IP incidence, studies were divided into high or low IP incidence groups. For the high IP incidence group, IP was associated with both temporary/permanent and permanent hypocalcemia. However, in the low IP incidence group, IP increased only the risk of temporary/permanent hypocalcemia and did not increase the risk of permanent hypocalcemia. As mentioned above, high IP incidence might be associated with some high-risk factors, such as a large extent of the procedure and a malignant pathologic diagnosis. Further analysis of temporary/permanent thyroidectomy cases found that IP influenced both temporary/permanent and permanent postoperative hypocalcemia. For malignant cases, IP increased only permanent hypocalcemia and did not increase temporary/permanent hypocalcemia. However, the majority of the 5 studies focusing on malignant cases were clinical hypocalcemia, and this should be kept in mind when interpreting this result.

There were several limitations in our study. First, the definition of permanent hypocalcemia varied between the included studies. Although we conducted subgroup analyses of different follow-up durations, the effect of other definitions, including continued calcium supplementation and serum calcium and serum parathormone levels, was not discussed due to insufficient sample size. Second, confounders and heterogeneity remained in our analysis. Surgery volume might also be a confounder in this topic because experienced high-volume surgeons might dissect more expertly and have greater anatomical knowledge of the parathyroid gland. In addition, thyroid pathology experts at higher-volume centers might be better at identifying IPs. However, few studies included relevant data. Some study included cases that received intraoperative parathyroid gland transplantation, and this a known factor affecting postoperative hypocalcemia. The influence of lymph node dissection and reoperation could also not be eliminated because is no recognized standard for reporting this topic. Some low-quality studies did not report data in a standardized fashion, which might also have caused the heterogeneity and publication bias found in our analysis. Further multicenter research might be necessary to address this issue. Sensitivity analysis, subgroup analysis, and a random-effects model were used to address this. The interpretation of our results should be based on a sufficient understanding of these limitations.

## Conclusion

This meta-analysis indicates that malignancy, central neck dissection, total thyroidectomy and reoperation are significant risk factors of IP. IP was found to increase the incidence of temporary/permanent and permanent postoperative hypocalcemia. We recommend more meticulous intraoperative identification of the parathyroid gland in thyroidectomy to reduce IP, particularly for total thyroidectomy and malignant cases. This study confirms the effect of IP on postoperative hypocalcemia and suggests that a new technique to help reduce IP is necessary.

## Supporting information

S1 Fig Funnel plot of temporary/permanent postoperative hypocalcaemia. Studies included in the review was shown by filled dots, while the missing studies was shown by hollow dots.
(EPS)

S2 Fig Funnel plot of permanent postoperative hypocalcaemia. Studies included in the review was shown by filled dots, while the missing studies was shown by hollow dots.
(EPS)

S3 Fig Sensitivity analysis of total postoperative hypocalcaemia. (EPS)

S4 Fig Sensitivity analysis of permanent postoperative hypocalcaemia. (EPS)

S5 Fig Location of incidentally resected parathyroid. (EPS)

S1 Table Main characteristic of studies excluded by full-text articles assessment. (DOCX)

S2 Table Incidence of total and permanent hypocalcaemia. (DOCX)

S3 Table Meta-regression analysis. (DOCX)

S4 Table Pathologic data of included studies. (DOCX)

S1 File PRISMA 2009 checklist. (DOC)

S2 File Raw data. (XLSX)

## Abbreviations

IP: incidental parathyroidectomy
RR: Risk Ratio
RD: Risk Difference
CI: Confidence Interval
