# Supplementary material for: Risk factors and outcomes of incidental parathyroidectomy in thyroidectomy: A systematic review and meta-analysis
Source: PLoS One. 2018 Nov 9;13(11):e0207088. doi: 10.1371/journal.pone.0207088 (PMC6226183; doi:10.1371/journal.pone.0207088)
Supplement: S1 Table — (DOCX) [file pone.0207088.s006.docx]

S1 Table Main characteristic of studies excluded by full-text articles assessment

| Author | Year | Location | Study type | Pathology | Procedure extent | Exclusion reason* |
| --- | --- | --- | --- | --- | --- | --- |
| Lee[30] | 1999 | USA | RC | Benign & Malignant | TT, LB | 2 |
| Lin[38] | 2002 | USA | RC | Benign & Malignant | TT, NT, LB | 3 |
| Pereira[39] | 2005 | Spain | RC | Malignant only | TT+CND | 3 |
| Rix[31] | 2006 | UK | RC | Benign & Malignant | TT, LB | 2 |
| Roh[40] | 2007 | Korea | RC | Malignant only | TT | 3 |
| Hsiao[41] | 2009 | Taiwan | RC | Malignant only | TT | 3 |
| Lee[26] | 2010 | Korea | RC | Malignant only | TT | 1 |
| Pesce[27] | 2010 | USA | RC | Benign only | TT | 1 |
| Campos[32] | 2012 | Brazil | RC | Benign & Malignant | TT, LB | 2 |
| Allios[33] | 2013 | Athens | RC | Benign & Malignant | TT | 2 |
| Abuelaish[34] | 2014 | Jordan | RC | Benign & Malignant | TT, LB | 2 |
| Garrahy[28] | 2014 | Ireland | RC | Benign & Malignant | TT, LB | 1 |
| Ozogul[42] | 2014 | Turkey | RC | Benign & Malignant | TT, LB | 3 |
| Yazici[35] | 2014 | Turkey | RC | Benign & Malignant | TT, LB | 2 |
| Kwan[29] | 2015 | Hong Kong | RC | Malignant only | TT, TT+CND | 1 |
| Merchavy[43] | 2015 | Canada | RC | Benign & Malignant | TT, CT | 3 |
| Hone[44] | 2016 | UK | RC | Benign & Malignant | TT, LB | 3 |
| Tartaglia[45] | 2016 | Italy | RC | Benign & Malignant | TT | 3 |
| Chew[46] | 2017 | Australia | RC | Benign & Malignant | TT | 3 |
| McGoldrick[36] | 2017 | Ireland | RC | Benign & Malignant | TT, LB, CT | 2 |
| Ozden[37] | 2017 | Turkey | RC | Benign & Malignant | TT, CT | 2 |

Abbreviation: RC, retrospective cohort; PC prospective cohort; TT total thyroidectomy; ST subtotal thyroidectomy; CT completion thyroidectomy; LB lobectomy; NT near-total thyroidectomy; CND central neck dissection; UT unilateral thyroidectomy; BT bilateral thyroidectomy.

* Exclusion reason: 1, unconvertible data; 2, lack of hypocalcemia data in control group; 3, definition of hypocalcaemia not clear.
