# Supplementary material for: Risk factors and outcomes of incidental parathyroidectomy in thyroidectomy: A systematic review and meta-analysis
Source: PLoS One. 2018 Nov 9;13(11):e0207088. doi: 10.1371/journal.pone.0207088 (PMC6226183; doi:10.1371/journal.pone.0207088)
Supplement: S2 Table — (DOCX) [file pone.0207088.s007.docx]

S2 Table Incidence of total and permanent hypocalcaemia

| Author | Year | Case, n | | IP incidence, % | Auto-transplantation rate, % | | Temporary/permanent hypocalcemia, n | | | | Permanent hypocalcaemia, n | |
| --- | --- | --- | --- | --- | --- | --- | --- | --- | --- | --- | --- | --- |
|  |  |  |  |  |  |  | Biochemical | | Clinical | |  |  |
|  |  | IP | Non-IP |  | IP | Non-IP | IP | Non-IP | IP | Non-IP | IP | Non-IP |
| Sasson[62] | 2001 | 21 | 120 | 14.9 | 10.0 | 18.0 | - | - | 1 | 9 | 0 | 1 |
| Sakorafas[61] | 2005 | 28 | 130 | 17.7 | 3.6 | 1.5 | 6 | 30 | 2 | 8 | 0 | 1 |
| Gourgiotis[13] | 2006 | 68 | 247 | 21.6 | 1.3* | | 15 | 57 | - | - | 0 | 0 |
| Abboud[47] | 2007 | 38 | 269 | 12.4 | 32.0 | 12.0 | 9 | 41 | 4 | 13 | - | - |
| Cavicchi[49] | 2007 | 60 | 534 | 10.1 | - | - | - | - | 23 | 112 | - | - |
| Irkorucu[51] | 2007 | 10 | 263 | 3.7 | 1.1* | | - | - | 1 | 56 | 0 | 0 |
| Page[14] | 2007 | 22 | 329 | 6.3 | 5.7* | | 7 | 128 | - | - | 0 | 6 |
| Sippel[64] | 2007 | 33 | 480 | 6.4 | - | - | 20 | 212 | 4 | 19 | - | - |
| Manouras[54] | 2008 | 100 | 408 | 19.7 | - | - | 3 | 13 | - | - | 0 | 0 |
| Erbil[50] | 2009 | 48 | 392 | 10.9 | - | - | 9 | 54 | - | - | - | - |
| Rajinikanth[60] | 2009 | 47 | 317 | 12.9 | - | - | 19 | 83 | - | - | 4 | 9 |
| Sorgato[8] | 2009 | 70 | 812 | 7.9 | 5.7 | 4.3 | - | - | - | - | 7 | 32 |
| Turanli[66] | 2009 | 25 | 392 | 6.0 | - | - | - | - | - | - | 7 | 25 |
| Ondik[56] | 2010 | 13 | 29 | 31.0 | - | - | - | - | 2 | 7 | 1 | 3 |
| Spiliotis[3] | 2010 | 32 | 283 | 10.2 | - | - | 10 | 11 | - | - | 2 | 1 |
| Youssef[9] | 2010 | 26 | 181 | 12.6 | 4.8* | | 2 | 16 | 2 | 6 | - | - |
| Khairy[10] | 2011 | 47 | 240 | 16.4 | - | - | 18 | 48 | - | - | 2 | 4 |
| Qasaimeh[59] | 2011 | 20 | 213 | 8.6 | - | - | 7 | 31 | - | - | 0 | 6 |
| Kalyoncu[52] | 2013 | 20 | 170 | 10.5 | - | - | 8 | 38 | - | - | 0 | 9 |
| Nair[55] | 2013 | 23 | 783 | 2.9 | - | - | 12 | 178 | - | - | 2 | 11 |
| Paek[12] | 2013 | 154 | 377 | 29.0 | 45.0* | | - | - | 52 | 83 | 10 | 9 |
| Sheahan[63] | 2013 | 12 | 114 | 9.5 | 10.3* | | 5 | 23 | 3 | 10 | 1 | 2 |
| Del Rio[16] | 2014 | 26 | 512 | 4.8 | - | - | - | - | - | - | 0 | 0 |
| Pergel[58] | 2014 | 23 | 433 | 5.0 | 10.7* | | - | - | - | - | 0 | 0 |
| Prazenica[17] | 2014 | 58 | 1010 | 5.4 | 6.9 | 7.4 | 22 | 185 | - | - | 1 | 15 |
| Song[65] | 2014 | 90 | 364 | 19.8 | 0 | 0 | - | - | - | - | 7 | 13 |
| Lee[53] | 2015 | 23 | 111 | 17.2 | 14* | | 4 | 23 | 8 | 17 | - | - |
| Wang[67] | 2015 | 123 | 315 | 28.1 | 8.7* | | - | - | - | - | 7 | 5 |
| Applewhite[48] | 2016 | 283 | 271 | 16.2 | - | - | 168 | 97 | 38 | 22 | - | - |
| Manatakis[11] | 2016 | 70 | 211 | 24.9 | 5.7 | - | 20 | 28 | 4 | 8 | 0 | 0 |
| Ozemir[57] | 2016 | 56 | 361 | 13.4 | - | - | 38 | 167 | - | - | 3 | 3 |
| Zhou[18] | 2016 | 78 | 308 | 20.2 | 33.2* | | - | - | - | - | 4 | 3 |
| Du[15] | 2017 | 35 | 306 | 10.3 | - | - | - | - | 10 | 124 | 0 | 1 |
| Lin[4] | 2017 | 204 | 2982 | 6.4 | 1.5 | - | 63 | 341 | - | - | 17 | 57 |
| Sitges-Serra[5] | 2017 | 47 | 123 | 27.6 | 28 | 23 | 30 | 56 | - | - | 7 | 4 |
| Total |  | 2033 | 14390 | 12.4 |  |  | 495 | 1860 | 154 | 494 | 82 | 220 |

Abbreviation: IP incidental thyroidectomy.

* There were not specific data of IP or non-IP in these studies. Thus we could only demonstrate the data of whole community.
