# Supplementary material for: Risk factors and outcomes of incidental parathyroidectomy in thyroidectomy: A systematic review and meta-analysis
Source: PLoS One. 2018 Nov 9;13(11):e0207088. doi: 10.1371/journal.pone.0207088 (PMC6226183; doi:10.1371/journal.pone.0207088)
Supplement: S3 Table — (DOCX) [file pone.0207088.s008.docx]

S3 Table Meta-regression analysis

|  | Covariates | Regression coefficient | SE | 95% CI for coefficient | P value |
| --- | --- | --- | --- | --- | --- |
| Temporary/permanent hypocalcemia | Continent, Asia | 0.618 | 0.7945 | -0.9393, 2.1753 | 0.4367 |
|  | Continent, Europe | 0.6338 | 0.7979 | -0.93, 2.1975 | 0.427 |
|  | Continent, America | 0.4485 | 0.8176 | -1.154,2.051 | 0.5833 |
|  | Year of study | 0.0265 | 0.0189 | -0.0105, 0.0636 | 0.1604 |
|  | NOS | 0.0217 | 0.082 | -0.1391, 0.1825 | 0.7911 |
|  |  |  |  |  |  |
| Permanent hypocalcemia | Continent, Europe | -0.0255 | 0.015 | -0.0550, 0.0040 | 0.0898 |
|  | Continent, America | -0.0148 | 0.0252 | -0.0641, 0.0345 | 0.5555 |
|  | Year of study | 0.0028 | 0.0019 | -0.0010, 0.0066 | 0.1455 |
|  | NOS | 0.0014 | 0.008 | -0.0143, 0.0171 | 0.8629 |

Abbreviation: SE, standard error; CI, confidence interval; NOS, Newcastle-Ottawa Scale.
