# Supplementary material for: Risk factors and outcomes of incidental parathyroidectomy in thyroidectomy: A systematic review and meta-analysis
Source: PLoS One. 2018 Nov 9;13(11):e0207088. doi: 10.1371/journal.pone.0207088 (PMC6226183; doi:10.1371/journal.pone.0207088)
Supplement: S4 Table — (DOCX) [file pone.0207088.s009.docx]

| Author | Year | No of IP | No of parathyroid | Parathyroid number | | | Intrathyroid  n(%) | Intracapsular  n(%) | Extracapsular  n(%) |
| --- | --- | --- | --- | --- | --- | --- | --- | --- | --- |
|  |  |  |  | one | two | three |  |  |  |
| Sasson[62] | 2001 | 21 | 26 | 17 | 3 | 1 | 13(50.0) | - | 13(50) |
| Sakorafas[61] | 2005 | 28 | 32 | 24 | 4 | - | 6(21.4) | 10(35.7) | 12(42.9) |
| Gourgiotis[13] | 2006 | 68 | 90 | 46 | 22 | - | - | - | - |
| Abboud[47] | 2007 | 38 | 43 | 33 | 5 | - | 9(23.7) | 15(39.5) | 14(36.8) |
| Irkorucu[51] | 2007 | 10 | 10 | - | - | - | 2(20.0) | - | 8(80.0) |
| Page[14] | 2007 | 22 | 22 | - | - | - | 5(22.7) | - | 17(77.3) |
| Rajinikanth[60] | 2009 | 47 | 50 | 44 | 3 | - | 21(44.7) | - | 26(55.3) |
| Sorgato[8] | 2009 | 70 | 70 | - | - | - | 11(15.7) | - | 59(55.3) |
| Turanli[66] | 2009 | 25 | 31 | 19 | 6 | - | - | - | - |
| Ondik[56] | 2010 | 13 | 13 | 13 | - | - | - | - | - |
| Spiliotis[3] | 2010 | 32 | 32 | - | - | - | 9(28.1) | - | 22(68.8) |
| Khairy[10] | 2011 | 47 | 54 | 41 | 5 | 1 | 24(44.4) | - | 29(53.7) |
| Qasaimeh[59] | 2011 | 20 | 23 | 17 | 3 | - | 5(21.7) | 5(21.7) | 13(56.5) |
| Kalyoncu[52] | 2013 | 20 | 24 | 16 | 4 | - | - | - | - |
| Song[65] | 2014 | 90 | 100 | 80 | 10 | - | - | - | - |
| Manatakis[11] | 2016 | 70 | 70 | 53 | 14 | 3 | 31(44.3) | 28(40.0) | 11(15.7) |
| Ozemir[57] | 2016 | 56 | 66 | 46 | 10 | - | - | - | - |
| Zhou[18] | 2016 | 78 | 91 | 66 | 11 | 1 | 12(13.2) | - | - |
| Du[15] | 2017 | 35 | 38 | 32 | 3 | - | 16(42.1) | - | 22(57.9) |
| Lin[4] | 2017 | 204 | 227 | 184 | 17 | 3 | 5(2.2) | 38(16.7) | 184(81.1) |
| Sitges-Serra[5] | 2017 | 47 | 52 | 42 | 5 | - | - | - | - |

S4 Table Pathologic data of included studies

Abbreviation: IP incidental thyroidectomy.
